# Kinesthetic imagery of musical performance

**DOI:** 10.3389/fnhum.2013.00280

**Published:** 2013-06-13

**Authors:** Martin Lotze

**Affiliations:** Functional Imaging, Institute for Diagnostic Radiology and Neuroradiology, Ernst Moritz Arndt University of GreifswaldGreifswald, Germany

**Keywords:** imagery, musicians, training, motor skills, multimodal integration

## Abstract

Musicians use different kinds of imagery. This review focuses on kinesthetic imagery, which has been shown to be an effective complement to actively playing an instrument. However, experience in actual movement performance seems to be a requirement for a recruitment of those brain areas representing movement ideation during imagery. An internal model of movement performance might be more differentiated when training has been more intense or simply performed more often. Therefore, with respect to kinesthetic imagery, these strategies are predominantly found in professional musicians. There are a few possible reasons as to why kinesthetic imagery is used in addition to active training; one example is the need for mental rehearsal of the technically most difficult passages. Another reason for mental practice is that mental rehearsal of the piece helps to improve performance if the instrument is not available for actual training as is the case for professional musicians when they are traveling to various appearances. Overall, mental imagery in musicians is not necessarily specific to motor, somatosensory, auditory, or visual aspects of imagery, but integrates them all. In particular, the audiomotor loop is highly important, since auditory aspects are crucial for guiding motor performance. All these aspects result in a distinctive representation map for the mental imagery of musical performance. This review summarizes behavioral data, and findings from functional brain imaging studies of mental imagery of musical performance.

## General introduction

Mental imagery of a piece of music in one's mind is commonly used by professional musicians for instance to rehearse difficult parts of an already executed musical passage (Lotze et al., 2003). Famous musicians like Vladimir Horowitz and Walter Gieseking reported frequent use of mental practice (Gieseking and Leimer, 1972; Schonberg, 1987). Mental music rehearsal includes different images of a musical piece: motor, somatosensory and auditory, but also emotional aspects. Most commonly mental imagery in musicians is related to reading the scores of a piece and mentally rehearsing predominantly the auditory aspects. However, this is only one of several aspects of mental rehearsal of a musical piece. In all, three aspects of mental imagery used by musicians have been differentiated (Repp, 2001; Keller, 2012).

Firstly, there is the silent reading of musical scores, requiring an advanced skill referred to as “notational audiation” (Brodsky et al., 2008). Secondly, there is action simulation during musical performance, including thinking of the ideal sound during performance, which might guide the movements but is also associated with the technique of anticipatory auditory imagery in playing in an ensemble (Keller, 2012). Thirdly, musicians perform mental practice away from the instrument.

We will concentrate here on this last aspect of mental rehearsal, and especially on the kinesthetic imagery technique. In brief, this review will present data on basic research on kinesthetic imagery. It will then overview the usage and the effects of kinesthetic imagery training. Since sensory input is so important in the training of musical expertise we will depict how multisensory and motor images might interact in musical imagery. We will then describe some mapping studies on kinesthetic imagery, imagery training and focus on mapping studies of kinesthetic imagery in instrument-talists and singers. The last part will deal with challenging developments in the research of kinesthetic imagery in musicians.

## Basic research on kinesthetic imagery and functional equivalence between motor execution and kinesthetic imagery

We know that musical imagery is multimodal but even very simple imagery tasks, where most research on imagery has been performed on, are multimodal, too. For instance, a simple imagined repetitive thumb tapping task, does include sensory feedback (somatosensory and auditory), motor imagery and imagery of temporal processes of tapping frequency. A typical instruction for this kind of kinesthetic imagery (or sensorimotor imagery) would be to first perform a certain movement repetitively and then go on doing it internally with prevention of actual movement. This example also illustrates that imagery following a period of prior experience of actual movement is widely used (e.g., Stinear et al., 2006). This is based on the assumption that motor imagery represents the result of consciously accessing the intention for a movement usually performed unconsciously during movement preparation (Jeannerod, 1994). A highly vivid conscious image of the movement might therefore most likely be accessible right after movement performance. Interestingly, good kinesthetic imagers have been identified as those who are able to selectively increase motor-evoked potentials over those muscles involved in the actual task being imagined, but not for those muscles that are not involved despite their nearby location (Lebon et al., 2012a). For finger tapping of the thumb only, the thumb muscle (opponens) but not the abductor of the fifth finger was increased in excitability for the good imagers, whereas the poor imagers also recruited the abductor digiti minimi. It is quite remarkable that both good kinesthetic imagers and professional musicians show parallels with respect to an increased focus on target muscles and decreased “enslavement” of neighboring muscles (Jerde, 2006).

There are several basic mechanisms which are common to both mental imagery of movements and their execution, as has been postulated by Jeannerod (2001). Firstly, the time taken to imagine the performance of a complex movement sequence is of similar duration to the movement execution itself (Bakker et al., 2008). However, very complex attention-demanding movements take longer to imagine than simple ones (Guillot and Collet, 2005). This is one indication that the process of imagination is not dependent only on the ability to execute a movement but also on central processing mechanisms. Another indication is that patients with lesions of the motor cortex and patients with Parkinson's disease show decreased movement velocity during both execution and imagery (Dominey et al., 1995), whereas patients with spinal lesions only show prolonged duration of execution—the duration of the imagery remains the same in this group (Decety and Boisson, 1990).

Secondly, physiological parameters, although not accessible voluntarily, are positively associated in executed and imagined movements with respect to observed changes in heart rate; increases in CO2-pressure and respiration frequency (Decety et al., 1991); and skin conductance responses (SCR) (e.g., Guillot et al., 2008). Decety (1996) proposed that during imagined activities a significant portion of the observed increase in autonomic response is of central origin. The authors interpreted this as an influence the mind exerts over the body, into believing that some movements are being executed. The third commonality between mental imagery of movements and their execution is the subjective rating of the mental effort to imagine a task and the fact that it is correlated with the amount of force needed for actual task execution (Decety and Lindgren, 1991).

## The usage and the effects of kinesthetic imagery training

As we have seen, motor imagery shows many parallels with motor execution with respect to physiological and behavioral parameters. All these findings point to the assumption that motor imagery could be based on the motor representations employed for actual movements. In fact several other data support this view. It has been shown for instance that the ability to imagine a movement is dependent on the posture of the body; incompatible postural signals affect imagery (Parsons, 1994). Imaging studies demonstrated that imagined and actual body position both influence the activity in neural structures during own-body simulation processes (de Lange et al., 2006).

Models of motor control provide a framework of the mechanisms by which the areas storing expertise in motor execution might be recruited and partially modified during kinesthetic imagery (Wolpert et al., 2003). An inverse model generates an appropriate motor command and the forward model maps the efference copy with the anticipated outcome of the action. The anticipated outcome might build a template against which the incoming information can be compared. Discrepancies between these require a rapid adjustment of the motor command and, on this basis, of the anticipated consequences of actions. Recently, a temporal framework of such a prior efferent copy has been postulated for the articulatory system: an auditory efference copy is presumably elicited approximately 170 ms after the somatosensory feedback from articulatory motor commands (Tian and Poeppel, 2010).

Overall, kinesthetic imagery might activate an internal model of a movement, which is dependent on the actual posture of the body. This involves an activation of a body representation in the reference space of the body itself and in relation to other objects. These spatial processes, providing a dynamic representation of the current postural configuration of the body utilised during movement planning and execution, are represented in the parietal lobe (e.g., Parkinson et al., 2010). As we will see later on, this area is quite important for kinesthetic imagery especially for more complex motor processes.

The idea that vivid kinesthetic imagery is based on experience in motor execution (Jeannerod et al., 1994) is in keeping with the reported positive relationship between expertise and imagery quality in athletes (Reed, 2002). The higher the expertise level, the more accurately a movement can be mentally rehearsed in tennis players (Fourkas et al., 2008); corticospinal facilitation of representation sites involved in actual task performance is only seen during imagery of the same tasks when the task has been previously trained actively. For imagery training these findings tell us that those athletes who have more detailed, more vivid and/or longer experience in motor execution are those who profit more from kinesthetic imagery training. Consequently, imagery techniques are most frequently applied for training in professional or high level athletes. It remains an open question whether this holds to be true for musicians, too. Imagery training is also frequently used for musical students and we know that there is an interference of the effect of training with the level of motor performance at the start of training. Those who start with a lower level are those who usually profit more.

It is also evident that mental training can be seen as a complementary technique to execution training but should not be used as a substitute to movement execution.

With respect to the training effect of kinesthetic imagery, it has been demonstrated that mental practice improves performance in athletes (Driskell et al., 1994). In addition, this technique has been shown to improve the dynamics of motor performance in a grapho-motor task (Yaguez et al., 1998) and the velocity of finger tapping movements (Lacourse et al., 2005). In musicians it has been demonstrated that mental rehearsal of the musical piece improves later performance (Theiler and Lippman, 1995).

Apart from the musicians group, it has even been demonstrated that training by using kinesthetic imagery improves the strength of an isometric movement (Ranganathan et al., 2004). Since no increase in muscle mass has been observed, the increase in strength may be caused by adaptive changes in the central processes. A decreased training effect of imagery compared to execution training may be caused by the lack of sensorimotor feedback, which might in turn explain the decreased progress in motor training in stroke patients (Floel et al., 2004). However, other authors developed training protocols, demonstrating that proper combinations of mental and physical training yield equal results in the same time than those applying physical training alone (Ross, 1985; Coffman, 1990). Furthermore, an increase of pitch accuracy was shown for mental practice compared to physical practice alone in guitarists. When using mental practice with a modeled recording of the music alternating with physical practice in guitar players, mental practice resulted in superior performance in tonal quality and memory coding in comparison to physical practice alone (Theiler and Lippman, 1995).

## Interaction of multisensory and motor images in musical imagery

Since the inverse model consists of both motor patterns and their sensory consequences, the content of musical imagery is multimodal and consists of a whole spectrum of kinesthetic (motor and somatosensory), but also auditory elements. Auditory aspects of imagery in musicians have been the topic of research for decades already (latest reviews: Halpern, 2012; Zatorre, 2012). The right auditory cortex seems to be of more importance than the left, since patients with removals of the right temporal lobe perform more poorly on both perception and imagery tasks compared with those with left temporal excision, and with controls (Zatorre and Halpern, 1993). When testing judgments of imagined musical pieces, such as pitch change (Zatorre et al., 1996), continuation of a melody (Halpern and Zatorre, 1999), musical timbres (Halpern et al., 2004), and tonal correctness of an imagined melody (Herholz et al., 2008), the involvement of different subunits of the bilateral auditory cortex, but also activation of other parts of the brain, such as the parietal lobe, have been identified. Overall, with more manipulations or transformations of the imagined known melody that are asked to be performed, there are more areas in addition to the auditory cortex that are involved in the task (Zatorre, 2012).

In the visual modality, the recruitment of primary areas during imagination has been shown to be highly correlated with the vividness of imagery (Cui et al., 2007).

Aleman et al. (2000) reported that musicians are not only better in musical mental imagery than non-musicians, but auditory musical imagery in general is increased in musically highly-trained subjects. However, other sensory imagery qualities, such as visual imagery capability, are not enhanced in musicians. Furthermore, it has been demonstrated that there is a positive association between the musician's auditory imagery abilities and success at learning novel piano pieces from notation in the absence of auditory feedback (Highben and Palmer, 2004). Use of auditory imagery during mental practice is associated with better post-practice performance (Bernardi et al., 2013).

## Mapping studies on kinesthetic imagery and imagery training

Mapping studies support the notion of a partial equivalence of motor execution and imagery as postulated before (Jeannerod, 2001). Functional maps measured during circumscribed and well-defined motor execution show a large overlap with those assessed during the same movement imagined (Stephan et al., 1995; Porro et al., 1996; Lotze et al., 1999; Munzert et al., 2008). In addition it has also been demonstrated that the more vivid imagery is, the more the motor pathways are recruited in a realistic way (Lorey et al., 2011).

Most overlap has been reported for the supplementary motor area (SMA), the premotor cortex (PMC), for parietal areas and for the cerebellum. In particular the posterior SMA and the PMC (BA 6) seem to be the predominant areas responsible for movement imagery. Neurons in the SMA are involved in the preparation of movements and it is reasonable that preparatory aspects of a movement may be closely related to motor imagery. The PMC, can be subdivided into a dorsal (dPMC) and a ventral (vPMC) area. Whereas the vPMC lies adjacent to the posterior part (BA44) of Broca's area in the left hemisphere and Broca's analog in the right hemisphere, the dPMC is more associated with anterior parts of the primary motor hand area (BA 4a). Different imagery strategies involve different parts of the PMC; where kinesthetic imagery involves the dorsal PMC, visual strategies involve more ventral parts (Binkofski et al., 2000). BA 44 activation has been described during imagery of targeted hand movements (Grafton et al., 1996). In addition, patients with left lateral prefrontal lesions are unable to imagine a motor task (Johnson, 2000), underlining the important functional role of this area and a functional lateralization for motor imagery. vPMC and BA 44 is the human representation of the mirror neural network, which represents internal sequence patterns of trained movements (Binkofski et al., 2000). In fact, these mirror neurons are increasingly active in musicians during training of new finger sequences, even when the procedure used in the fMRI-experiment is not directly associated with the instrumental context performed in previously (Pau et al., 2013). When hands were visually presented performing guitar chords, the mirror neural system was more active in guitar players than in musically untrained subjects (Vogt et al., 2007). In conclusion, kinesthetic imagery and movement observation share functional resources located in the ventral PMC which seem to represent motor engrams of complex movement patterns.

The primary motor cortex is ~50–70% less involved [blood oxygen level dependent (BOLD) -effects magnitude] during imagery than during execution of the same movement. Most of the studies describing primary motor cortex activation during kinesthetic imagery used an instruction method for mental imagery, with the executed movement preceding imagined movement. The studies controlled the execution of the movements via the use of electromyography (EMG) of the effector muscles (e.g., Lotze et al., 1999). Interestingly, the primary motor cortex is increasingly involved in more complex imagined movements (Kuhtz-Buschbeck et al., 2003).

Remarkably, the cerebellum is also activated during imagery of simple hand movements (Decety et al., 1994) although no actual sensorimotor feedback is present during imagery. A closer look revealed distinct areas activated during imagery compared with those active during motor execution; activation during imagery is located more posterior-inferior (centered in Larsell's lobule HVII) than that described during actual movements (centered in Larsell's lobule HIV; Lotze et al., 1999). It has been assumed that the decrease of activation in the anterior cerebellum during imagery is due to missing afferent information. The anterior cerebellar hemisphere is predominantly active during sensorimotor exploration movements (Gao et al., 1996) and receives sensory information via the spinocerebellar tract. Information about cortical control of movement is provided by the corticopontino-cerebellar tract, which is closely connected via the ventral part of the nucleus dentatus to the dorsolateral prefrontal parts of the cortex (Middleton and Strick, 1994). This tract closely links the upper part of the posterior cerebellum to the SMA and the PMC. Along this pathway, aspects of movement coordination— but also inhibition of movement execution— may be connected between the SMA and the posterior cerebellar hemisphere. This inhibitive pathway might well-suppress actual movement execution in the experimental setting of kinesthetic imagery. This setting includes a short sequence of movement execution and instructs the participants to avoid actual EMG-responses in target muscles in the imagery condition that follows (see also the previous chapter on basic characteristics of kinesthetic imagery research).

However, this might only be one network inhibiting motor execution during imagery. It has been proposed recently (Guillot et al., 2012) that inhibition of execution during mental imagery might take place in a network of different central representation areas, including parietal (Schwoebel et al., 2002), brain stem or cerebellar (Lotze et al., 1999) areas, or in prefrontal—basal ganglia circuits.

Cerebellar activation seems to be also interesting with respect to the cerebellum's role for forward processing of movement and fast regulation of movement control dependent on sensorimotor feedback (Imamizu et al., 2000). Whereas feedback is lacking during imagery, prospective mechanisms might be recruited in different interconnected areas which predominantly represent the following functions: vPMC for motor pattern storage; SMA for movement ideation and sequencing; the medial cingulate cortex for attention; and the posterior cerebellar hemisphere for processing an additional control loop of movement inhibition and motor sequencing. It is also important to mention that the cerebellar hemispheres do have a role in timing and the estimation of duration (Ivry et al., 2002), which might be a necessary feature for parallel processing. Interestingly, these forward models in the cerebellar hemispheres might also be involved in context- specific activations, as is the case in instrument-specific sensorimotor loops (Gebel et al., 2013).

For more complex motor imagery and sensorimotor integration, the superior parietal lobe is highly important; patients with parietal lesions were found to have problems predicting the time necessary to perform differentiated imagined finger movements and visually-guided pointing gestures (Sirigu et al., 1995). It has been shown that the parietal lobe is interconnected with the primary motor cortex during mental imagery of simple hand movements (Lebon et al., 2012b). A suppression of parietal-M1 interaction has been detected during kinesthetic imagery, underlining the inhibitive role of the parietal lobe during imagery. It is highly interesting whether different regions in the parietal lobe code for different aspects of imagined movement performance or movement inhibition, coding for the spatial qualities of the movement, and the access to the storage of the movement trajectory. In addition, it has been hypothesized that motor intention is represented in the posterior parietal lobe, and the same place has been proposed as being responsible for the evaluation of an efference copy and the prediction of movement (Desmurget and Sirigu, 2009). Recent magnetoencephalographic results showed that during both movement execution and imagery, a posterior parietal dipole near the anterior intraparietal sulcus can be identified. This was at its maximum 90 ms before the execution latency (Tian and Poeppel, 2010). Overall, the recruitment of functional areas is dependent on several factors, such as concrete content of imagery (Solodkin et al., 2004), the perspective of imagery (Lorey et al., 2009), the imagined movement effector (Stippich et al., 2002), the position of the body during imagery (Lorey et al., 2009), different levels of demand for movement precision (Lorey et al., 2010), and the subject's ability to imagine (Guillot et al., 2008). For instance, first-person perspective increases left hemispheric motor representation in comparison to a third-person view (Ruby and Decety, 2003). While there is extensive research on modulation of motor areas by motor execution, studies on modulation with imagery quality are less common. One study investigated the effect of precision on the functional representation of kinesthetic imagery of grip movements, demonstrating that imagery of particularly precise movements is processed in the anterior cerebellar hemisphere and superior parietal lobe (Lorey et al., 2010).

## Mapping studies of kinesthetic imagery in instrumentalists and singers

In contrast to mapping studies on imagery in general—and on kinesthetic imagery mapping—studies on musical imagery are rare and mostly unspecific. This might be due to the largely holistic approach typically applied for investigating imagined musical performance. However, it can be criticized that more specific paradigms need to be tested if we are to understand how different aspects of imagery might be modulated. Overall, during imagined musical performance a wide network of brain activation can be assumed. Furthermore, sensory coactivation might be increasingly involved not only for the somatosensory cortex. In addition, since some professional musicians have more experience in movement performance patterns, a high contribution of vPMC can be assumed in these subjects.

Training of finger sequences on a piano for 2 h over a period of 5 days, with both movement imagery and movement execution, results in a substantial performance gain. Furthermore, the representation areas of long finger flexors/extensors in the contralateral primary motor cortex, as assessed with transcranial magnetic stimulation (TMS), is increased (Pascual-Leone et al., 1995). Movement imagery alone also results in a training effect, but a combination of imagery with execution training displays a greater increase in performance. Most interestingly the imagery group demonstrated the same training effect after one additional execution training session as the execution group, highlighting the importance of combining imagery and movement execution in musical performance training.

Contrary to these findings on the involvement of the primary motor cortex in kinesthetic imagery tasks (for a review see Lotze and Zehntgraf, 2010), Langheim et al. (2002) investigated imagined musical performance, and did not find cerebral activations in the contralateral primary motor cortex. Instead, they reported an activated network of lateral cerebellar, superior parietal and superior frontal activation and concluded that this network is likely to coordinate the complex spatial and timing components of musical performance. There seemed to be a considerable overlap of representation sites reported for forward model representation and reports on the representation of an internal motor engram with the findings of the Langheim et al. (2002) study.

Meister and colleagues compared activation maps measured with fMRI assessed during right-hand keyboard execution and imagery of a Bartok piece in musical students. They found a bilateral frontoparietal network during the imagery task without a significant contribution of the primary motor cortex (Meister et al., 2004).

**Figure 1:**
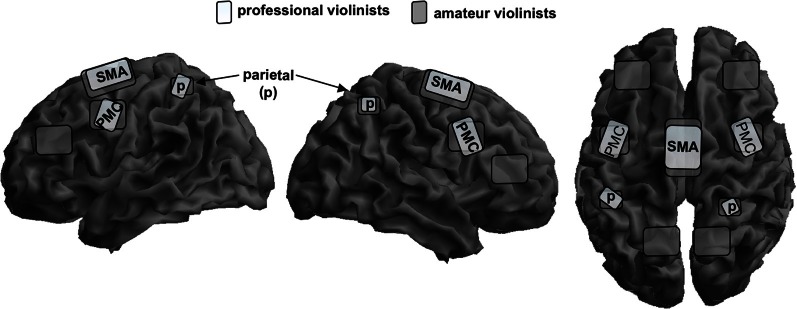
**Schematic overlay of mental rehearsal of playing a violin concerto only with the left hand in professional violinists (bright gray) and amateur violinists (dark gray) on a segmented gray matter cortex**. Professionals focus their activation patterns on the bilateral superior parietal cortex, p; bilateral premotor cortex, PMC; and to a lower magnitude on the supplementary motor area, SMA. Amateurs show widely distributed bilateral representation sites in the same areas but also the posterior parietal lobe and the lateral prefrontal cortex. Cerebellar and subcortical representation is not shown here. This schema is based on data from Lotze et al. (2003).

We investigated fMRI-activation maps in a group of professional and amateur violinists during imagined musical performance of the first passage of Mozart's violin concerto in D-Major (Lotze et al., 2003; for an schematic overview see Figure 1). Professional violinists scored higher in the vividness of movement imagery when compared with the amateurs. Rhythm and pitch imagination scores correlated positively with lifetime and weekly training. With respect to the functional imaging results, professionals showed increased activations during imagery in the right vPMC and the left anterior cerebellar hemisphere in the representation areas of the fingers (Larsell's lobule HVI). An increased activation in the anterior ipsilateral cerebellar regions of finger representation in the professional group may illustrate more efficient recruitment of stored sensorimotor engrams during motor imagery. In contrast, amateurs showed increased representation in the anterior SMA consistent with increased effort for sequencing finger patterns even when only imaging them. Moreover, whereas all musicians reported using imagery techniques for training in Langheim et al.'s study, only the professional group did in the Lotze et al. (2003) study. The professional group showed a negative association between the magnitude of contralateral (right) M1-activation during imagined violin playing (with the left hand only) and their self-assessment of performance improvement through the use of imagery techniques. Given that only those musicians who experience benefits from mental rehearsal of the musical piece actually employ imagery in their training, it could be assumed that with increasing use of imagery, musicians exhibit decreased activity within the right precentral gyrus, thereby reducing motor attention. In accordance with the observations of Langheim et al. (2002) and earlier studies, the right primary auditory cortex was not activated during imagery of musical performance. The lack of primary auditory and motor activation might also be associated with a decrease of activation magnitude generally seen after extensive training. Representation might then be centered in those areas representing motor engrams or internal models of movement processing (see also Pau et al., 2013).

**Figure 2:**
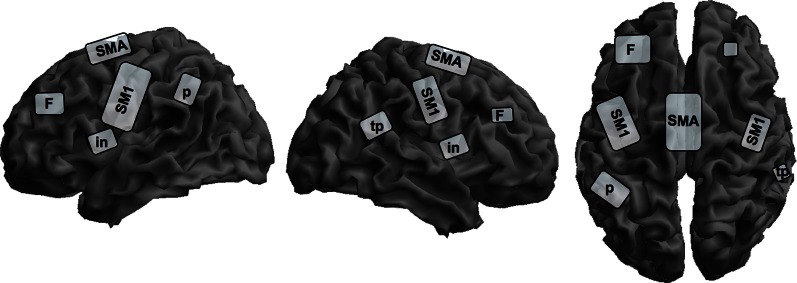
**Schematic overview of cortical representation of mental rehearsal of singing an Italian aria in singers with different levels of professionalism on a segmented gray matter cortex**. Imagined singing shows cortical activation in the bilateral primary sensorimotor cortex, SM1; insula, in; prefrontal lobe, F; left inferior parietal lobe, p; and right parietotemporal junction, tp; and the supplementary motor area, SMA. Subcortical activation sites in the limbic system, basal ganglia, thalamus, brain stem, and cerebellum are not depicted on the cortical surface. This schema is based on data from Kleber et al. (2007).

There are several studies investigating covert singing because of problems with artifacts during actual singing in an fMRI-scanner, but only one study investigated professional singers during imagined singing (Kleber et al., 2007; for an schematic overview see Figure 2). Singers of differing professional levels were scanned with fMRI; they sang parts of an Italian aria and imagined singing the same piece.

Cerebral activation sites during imagined singing were centered in a fronto-parietal network including motor areas (SMA, PMC), Broca's area and its homologue (no lateralization) and the superior and inferior parietal lobe. Additionally, subcortical motor areas in the cerebellum and basal ganglia, and the midbrain were involved. In contrast to studies with instrumentalists, we observed significant primary motor and somatosensory cortex activation and thalamus activation during imagined singing. Interestingly, areas processing emotions also showed intense activation (anterior cingulate cortex and bilateral insula, hippocampus, and anterior temporal poles, bilateral amygdala). This is quite remarkable, since to our knowledge no functional imaging study on professional instrumentalists has ever reported emotional areas being active in processing musical performance or kinesthetic imagery. Comparable to instrumentalists imagining playing a musical piece, singers showed no activation in the primary auditory cortex or in the auditory belt area, but in the temporo-parietal lobe bilaterally. This result is in line with several observations on sound and music imagery, which found only activity in auditory association cortices, but not the primary auditory cortex (Halpern and Zatorre, 1999; Yoo et al., 2001; Ducreux et al., 2003; Kraemer et al., 2005; Zatorre and Halpern, 2005). It is interesting in this regard that our subjects rated the vividness of imagined singing as high and reported no problems in attention during the imagery task. Additionally, since we applied a sparse sampling technique, distraction by the scanner noise was avoided.

For instrumentalists activation of the primary auditory cortex (A1) could not be demonstrated during imagined musical performance (Langheim et al., 2002; Lotze et al., 2003). However, A1 is active when musicians are tapping a trained musical sequence without any auditory feedback. We therefore argue that an automatized loop between M1 and A1 is only triggered if one of the two conditions is actually present: motor performance associated with auditory feedback (Gebel et al., 2013) or auditory presentation associated with trained motor performance (D'Ausilio et al., 2006). If neither of these interactions is present, the loop between the primary auditory and motor cortex is not activated.

An auditory association area in the left hemisphere was reported to be responsible for the control of spoken and listened words (Hickok et al., 2003). We also observed activity around the peak of this area in the temporo-parietal lobe during both active and imagined singing, although it was bilaterally expressed. It is possible that auditory association for leading the melody, during both singing and imagined singing, may be represented in this area.

We have already mentioned that kinesthetic imagery of a musical piece does not only involve audio-motor networks but also other cognitive strategies (e.g., memorizing the score in its temporal sequencing and expression) involved in recalling a musical piece (Halpern, 2012). Additionally, for a concert, imagery of the interaction with other instrumentalists or singers of the ensemble is of importance (Keller, 2012). Scientifically, these processes are quite difficult to control for; the scientist is always in a conflict between investigating true-to-life mental rehearsal of musical practice without control of single components, and the separation of single elements which he/she thinks are part of mental rehearsal in musicians.

## Challenging developments in the research of kinesthetic imagery in musicians

Modern data evaluation strategies enable us to record real world data in groups of musicians interacting, as has been nicely demonstrated in the kinematic studies of ensembles in the Keller laboratory (Keller, 2012). These real world observations are of high value. On the other hand, interesting data on the increased capability of musicians can also be observed in highly controlled experiments, where a transfer of trained knowledge on new tasks is measured (e.g., Pau et al., 2013). It is an open question whether highly experienced musicians, as it has been demonstrated for athletes, do profit more from imagery training than novices. This might be a promising experiment to perform. In particular, experiments on increased focal excitability of the motor cortex in musicians trained in kinesthetic imagery (see Lebon et al., 2012a,b)—even when only sounds which are usually associated with motor recruitment during play are presented (D'Ausilio et al., 2006)—might be a further step to understand specific changes after imagery training in musicians. Overall, we are not yet in a position to suggest specific training protocols for students in music based on the neurophysiologic investigations in imagery.

## Conclusion

Systematic research into the neurophysiological correlates of mental rehearsal of musical motor performance is in a very basic state. In order to suggest practical applications for training with kinesthetic imagery from the experience of neurophysiologic studies, more behavioral protocols on the effects on different imagery strategies for improving musical performance have to be tested. To develop strategies for improving the use of imagery training in musicians or for testing and selecting appropriate imagery techniques for training, an interaction of experienced musicians and neuroscientific research is essential. The most fruitful advances might be those, applying cognitive tests to identify characteristic performance gains dependent on different training procedures. Additionally, the control of mental processes performed during mental imagery is essential. The following questions might lead us: How can we identify good imagers? Why and how do subjects profit from imagery training? Is the temporal duration of imagined performance and vividness rating enough or shouldn't we apply more sophisticated neurophysiological tests (SCR, MEP, EEG) to control and investigate the quality of imagery? All these questions might help to understand the fascinating processes associated with experienced performance of musicians.

### Conflict of interest statement

The author declares that the research was conducted in the absence of any commercial or financial relationships that could be construed as a potential conflict of interest.
